# Increased IL-6 secretion by aged human mesenchymal stromal cells disrupts hematopoietic stem and progenitor cells' homeostasis

**DOI:** 10.18632/oncotarget.7690

**Published:** 2016-02-24

**Authors:** Kelsey O'Hagan-Wong, Stéphanie Nadeau, Audrey Carrier-Leclerc, Felipe Apablaza, Reggie Hamdy, Dominique Shum-Tim, Francis Rodier, Inés Colmegna

**Affiliations:** ^1^ Division of Rheumatology, Department of Medicine, McGill University, Montreal, QC, Canada; ^2^ CRCHUM and Institut du Cancer de Montréal, Montreal, QC, Canada; ^3^ Shriners Hospital for Children and Division of Orthopedic Surgery, McGill University, Montreal, QC, Canada; ^4^ Divisions of Cardiac Surgery and Surgical Research, McGill University, Montreal, QC, Canada; ^5^ Department of Radiology, Radio-Oncology and Nuclear Medicine, Université de Montréal, Montreal, QC, Canada

**Keywords:** mesenchymal stromal cells, hematopoietic stem and progenitor cells, aging, senescence, senescence-associated secretory phenotype, Gerotarget

## Abstract

Hematopoietic stem and progenitor cell (HSPC) homeostasis declines with age, leading to impaired hematopoiesis. Mesenchymal stromal cells (MSC) are critical components of the bone marrow niche and key regulators of the balance between HSPC proliferation and quiescence. Accrual of DNA damage, a hallmark of cellular aging, occurs in aged MSC. Whether MSC aging alters the bone marrow niche triggering HSPC dysfunction is unknown. Using a human MSC-HSPC co-culture system, we demonstrated that DNA damaged MSC have impaired capacity to maintain CD34^+^CD38^−^ HSPC quiescence. Furthermore, human MSC from adult donors display some hallmarks of cellular senescence and have a decreased capacity to maintain HSPC quiescence and the most primitive CD34^+^CD38^−^ subset compared to MSC from pediatric donors. IL-6 neutralization restores the MSC-HPSC crosstalk in senescent and adult MSC-HSPC co-cultures highlighting the relevance of the local microenvironment in maintaining HSPC homeostasis. These results provide new evidence implicating components of the MSC secretome in HSPC aging.

## INTRODUCTION

Hematopoietic stem and progenitor cells (HSPC) sustain the normal turnover of blood cells throughout an individual's lifetime. The regulation of HSPC function is a complex process that involves the integration of HSPC inherent programs with signals originating from components of the bone marrow (BM) niche [1]. These signals ensure the balance between HSPC quiescence, to protect the stem cell pool from premature exhaustion, and HSPC cycling which is required to accomplish efficient hematopoiesis [2].

Central to the regulation of HSPC quiescence are the multifaceted interactions of HSPC with key cellular components of the BM niche, among them multipotent mesenchymal stromal cells (MSC) [3]. MSC contribute to the HSPC microenvironment through complex cell-cell interactions and the secretion of hematopoietins that regulate HSPC proliferation, differentiation, and migration [3, 4, 5, 6].

Recent evidence in myelodysplastic syndromes suggests that BM-MSC senescence could impact HSPC homeostasis [7] and that targeting senescent cells in animal models rejuvenate aged HSPC [8]. Cellular senescence is a vital tumor suppression mechanism also involved in aging and tissue repair [9]. The accumulation of senescent cells in premature aging models directly contributes to age-associated organ dysfunctions [6], probably owing to their senescence-associated secretory phenotype (SASP), a potent pro-inflammatory microenvironment remodeler [10]. Whether MSC senescence during aging impacts the bone marrow microenvironment remains undefined. Given that DNA damage is a hallmark of cellular senescence and aging [9, 11], we tested the impact of MSC irradiation-induced cellular senescence on the modulation of HSPC homeostasis. We demonstrate that DNA damage induces a classical senescent phenotype in MSC including the production of high levels of IL-6 that in turn mediates the reduced capacity to preserve HSPC quiescence and to maintain immature CD34^+^ subpopulations. Furthermore, our data suggest that MSC from older donors, despite of not harboring a complete senescent phenotype behave functionally as senescent cells secreting increased levels of IL-6 that alters HSPC biology.

## RESULTS

All adipose derived MSC used in this study met the minimal criteria proposed by the ISCT [12] including a spindle shaped morphology and the expression of positive MSC markers in more than 95% of cells (CD73 CD105, and CD90) without lineage commitment markers (CD34 CD19 CD14 and HLADR) (Supplementary Figure 1A). At the end of passage 3, all cells differentiated *in vitro* to osteoblasts, adipocytes and chondrocytes (Supplementary Figure 1B).

### DNA damage induces MSC senescence

**Figure 1:**
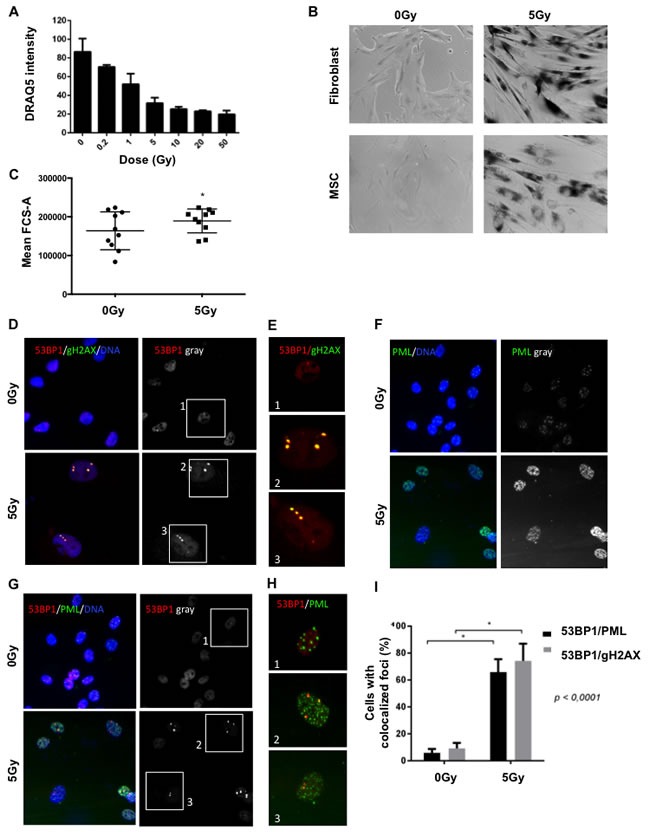
Irradiation induces senescence biomarkers in MSC **A.** MSCs were exposed to increasing doses of radiation (0 - 50 Gy) and proliferation was assessed 8 days later using LI-COR analysis (DRAQ5 fluorescence intensity (total DNA content). **B.** Senescence-associated beta-galactosidase activity was assessed at baseline and 9 days following 5 Gy irradiation in fibroblasts (positive controls, HCA2-hTERTs) and MSCs. **C.** MSC cell size was evaluated 8 days following 5 Gy irradiation using FACS. **D.** Direct molecular markers of DNA double strand breaks were evaluated in control and irradiated MSC using immunofluorescence (9 days following irradiation with 5 Gy). Representative images of cells harboring small nuclear DNA damage foci constituted of both 53BP1 (red) and phospho-H2AX (green) (yellow highlight red-green colocalization, the nuclei are counterstained in blue (DAPI)). The extracted 53BP1 red channel is presented in grayscale to highlight 53BP1 DNA damage foci that appears like nuclear white dots (right panels). Selected cells are boxed in white squares labeled 1-3 for magnification in **E.** to highlight colocalization between 53BP1 (red) and phospho-H2AX (green). Note the yellow dots in irradiated cells (box 2 and 3). **F.** The molecular biomarker of senescence (PML, green) was evaluated in control and irradiated MSC using immunofluorescence (nuclei counterstained in blue (DAPI)). Notice increased total levels of PML and PML nuclear bodies in irradiated cells. The extracted PML green channel is presented in grayscale to highlight the nuclear increase in PML levels (right panels) **G.** Representative images of cells harboring DNA-SCARS highlighted by 53BP1 (red) and PML (green) colocalization. Nuclei are counterstained in blue (DAPI). The extracted 53BP1 red channel is presented in grayscale to highlight 53BP1 DNA damage foci that appears like nuclear white dots (right panels). Selected cells are boxed in white squares labeled 1-3 for magnification in **H.** to highlight colocalization (yellow) between 53BP1 (red) and PML (green). Note the yellow dots in irradiated cells (box 2 and 3). **I.** The quantified data for the representative images presented for DNA damage foci (53BP1-gH2AX colocalization) and DNA-SCARS (53BP1-PML colocalization) is presented as the percentage of cells with > 1 colocalized nuclear foci. Mean ±SD of 4 independent experiments are reported where (*) represents *p* ≤ 0.0001.

We have previously shown that DNA double strand breaks induce hallmarks of cellular senescence in human fibroblasts and endothelial cells including the activation of a persistent DNA damage response (DDR) [13, 14]; and that the accumulation of DNA damage is a salient feature of cellular aging [9, 11]. To determine whether DNA damage is sufficient to drive a complete cellular senescence phenotype in MSC, we first treated MSC with escalating doses of gamma irradiation and determined whether these cells underwent permanent growth arrest, a hallmark of cellular senescence [11]. MSC proliferation was evaluated 8 days post irradiation using quantitative LI-COR analysis of total cellular DNA (Figure 1A). We observed a dose-dependent decrease in proliferation that leveled around 5Gy, suggesting that this represents the minimal dose that generated prolonged senescence-like MSC growth arrest. To characterize other senescence-associated hallmarks in MSC irradiated with 5 Gy, we first measured senescence-associated beta galactosidase (SABG) activity, the most widely used senescence biomarker. SABG activity was detected in damaged MSC or fibroblasts 9 days following irradiation while no detectable SABG expression was found baseline (0 Gy) (Figure 1B). We also observed that irradiated MSC lost their spindle shape morphology and acquired a typical senescence-associated enlarged and flattened phenotype. This was confirmed by a significant increase in cell size 8 days after irradiation as measured by flow cytometry (Figure 1C).

### Irradiated MSC express molecular markers of cellular senescence

Cellular senescence is characterized by a series of hallmarks including the activation of a persistent DDR [11, 14, 15]. Following DNA double-strand break (DSB), a series of chromatin modifications triggered by the DDR kinase ATM can be visualized in the damaged nuclei as DNA damage foci. Markers of DNA damage foci include the local phosphorylation of histone variant H2AX at serine 139 residue (γH2AX) and the recruitment of the damaged chromatin reader 53BP1 (p53 binding protein 1) [13, 16]. We performed immunofluorescence on non-irradiated controls and irradiated MSC and readily observed the appearance of colocalized nuclear γH2AX/53BP1 foci in irradiated cells (Figure 1D-1E). The tumor suppressor PML (promyelocytic leukemia) is also part of the DDR and an important modulator of p53 that is consistently up-regulated in senescent cells [17]. Immunoflurescence targeting PML revealed a strong increase of the protein in MSC, 10 days after 5Gy irradiation (Figure 1F). When DNA damage foci remains unrepaired for more than 48 hours following damage induction, they are converted to a structure termed DNA-SCARS, which is revealed by their juxtaposition to another nuclear domain, the PML nuclear bodies or PML-NBs [14]. We evaluated the presence of DNA-SCARS in irradiated MSC by detecting co-localization between γH2AX or 53BP1 and PML-NBs and found a consistent increase in damaged cells (Figure 1G-1H and summary data in Figure 1I). Overall, irradiated MSC display all the critical hallmarks associated with the presence of a senescence-associated persistent DDR.

**Figure 2:**
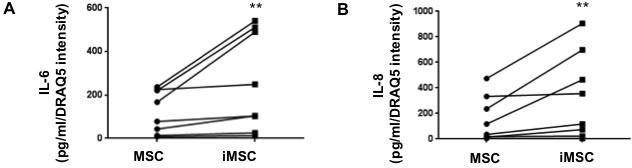
Increased secretion of senescence-associated cytokines by irradiated MSC The production of IL-6 and IL-8 in iMSC (5Gy) and controls was evaluated by ELISA 9 days after irradiation. iMSC produced significantly higher levels of IL-6 **A.** and IL-8 **B.** compared to controls. Mean (triplicate) of *7* independent experiments are reported where (**) represents *p* ≤ 0.01.

Finally, to evaluate the ability of DNA damage-induced senescent MSC to impact the microenvironment, we measured the secretion of two highly conserved components of the SASP: the pro-inflammatory cytokines IL-6 and IL-8 (Figure 2) [10]. The secretion of IL-6 and IL-8 by MSC consistently nearly doubled (*p* = 0.004) after irradiation although there was a large variability in the baseline cytokine production associated to individual donors. Overall the fold increase for IL-6 secretion in all paired samples (pre-IR *vs*. post-IR) ranged from 1.11 to 2.92 for an average increase of 1.95 fold while IL-8 secretion increased within a range of 1.07 to 5.40 for an average of 2.61 fold.

Thus, irradiation-induced DNA damage promotes a classical cellular senescence phenotype in MSC including the expression of a SASP [10, 13, 18].

**Figure 3:**
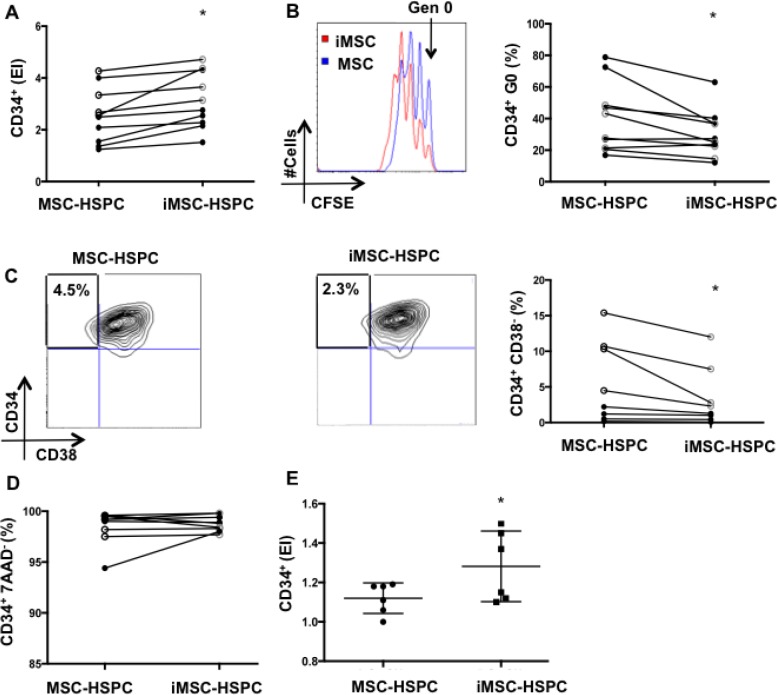
Senescent MSC reduce HSPC quiescence and the frequency of primitive CD34+CD38- cells **A.** CD34^+^ expansion, **B.** percentage of CD34^+^ in generation 0, **C.** frequencies of CD34^+^CD38^−^ subpopulations, and **D.** CD34^+^ viability were evaluated in MSC-HSPC and iMSC-HSPC (5Gy) co-cultures (1:1 ratio) following 4 days of co-culture. Open-faced circles represent MSC from pediatric donors (< 16 years-old) and black circles represent MSC from adult donors (> 40 years-old). **E.** CD34^+^ proliferation was also evaluated following HSPC culture with conditioned medium from MSC and iMSC. Mean ±SD of 6-10 independent experiments are reported where (*) represent *p* ≤ 0.05.

### MSC senescence leads to decreased HSPC quiescence *in vitro*

Senescent cells have the capacity to modulate their microenvironment [10, 11]. Given the critical role that MSC play in preserving normal HSPC function over time, we sought to investigate how MSC senescence might impact their capacity to support HSPC in *vitro*. To test this we co-cultured HSPC for 4 days with either non-irradiated MSC (controls) or irradiated MSC (senescent or iMSC) (Figure 3). To control for MSC death and to ensure that equal numbers of MSC were plated in irradiated and non-irradiated co-culture conditions, MSC were counted and re-plated 48 hours following irradiation.

Senescent MSC induced significantly higher CD34^+^ expansion compared to controls (expansion index 3.12±1.31 *vs*. 2.56±1.21, *p* = 0.002) (Figure 3A). The analysis of CD34^+^ proliferation also revealed that senescent MSC maintained a significantly lower proportion of CD34^+^ in generation 0 compared to non-irradiated MSC (33.11±22.35 *vs* 41.25±26.70, *p* = 0.01) (Figure 3B). Moreover, senescent MSC maintained a significantly lower proportion of CD34^+^CD38^−^ cells compared to non-irradiated MSC (3.99± 1.8 *vs*. 7.37±v 5.99, *p* = 0.02) (Figure 3C). Of relevance, CD34^+^ viability was not different (*p* = 0.4) in irradiated and non-irradiated MSC-HSPC co-cultures (Figure 3D). To test whether soluble factors mediated the increased HSPC expansion in iMSC-HSPC co-cultures, we performed supernatant transfer experiments. Conditioned medium from senescent MSC induced significantly higher HSPC expansion compared to conditioned medium from control MSC (1.1±0.1 vs. 1.3±0.2) (Figure 3E).

Largely, these results imply that MSC senescence induced by DNA damage alters the MSC capacity to support HSPC *in vitro* at least in part *via* secreted factors. Specifically, senescent MSC are less capable to maintain CD34^+^ quiescence and to preserve the CD34^+^CD38^−^ subpopulation.

### MSC from adult donors display senescence markers

**Figure 4:**
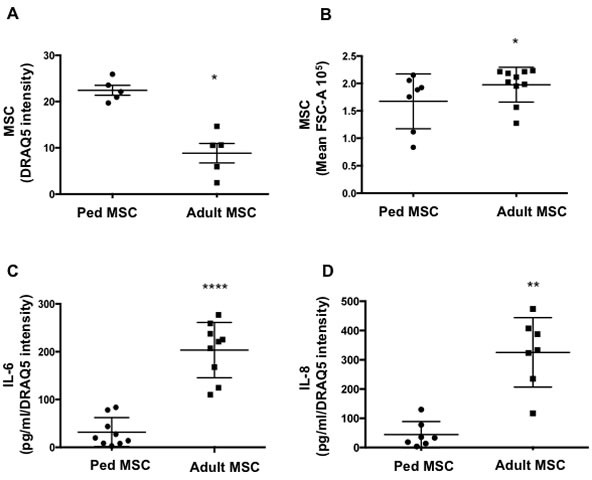
MSC from adult donors have a senescence-like phenotype **A.** MSC were isolated from adult (> 40 years) and pediatric donors (< 16 years). MSC proliferation was measured by DRAQ5 intensity day 4 post seeding in adult and pediatric samples. **B.** Cell size was measured by flow cytometry (Mean FSC-A) in adult and pediatric MSC samples. **C.** IL-6 and **D.** IL-8 production by adult and pediatric MSC were quantified by ELISA. Mean ±SD of 7-10 independent experiments are reported where (*) represents *p* ≤ 0.05.

The wide range of inflammatory cytokine secretion by non-irradiated MSC samples (Figure 2) suggested a potential association with the donors' age. To determine whether MSC undergo cellular senescence as part of the normal aging process *in vivo*, we comparatively examined the expression of senescence biomarkers in passage 3 (P3) MSC isolated from pediatric (age < 16 years) and adult (age > 40 years) patients. Adult MSC not only had a larger size but also displayed reduced proliferation rates compared to their pediatric counterparts (Figure 4A-4B). Probing the SASP also revealed that adult MSC produced significantly higher levels of the senescence-associated cytokines IL-6 and IL-8 compared to pediatric MSC (Figure 4C-4D). In fact, the difference in IL-6 or IL-8 (*p* = 0.0001) secretion between adult and pediatric MSC was much higher than what was observed in any samples following irradiation (IL-6 increase in adults: 5.46 fold; IL-8 increase 5.89 fold). However, consistent with what was previously reported in human bone marrow [19] and adipose MSC [20] in elderly individuals, we found low to undetectable levels of SABG (below 10%) in all early passage cultures tested, irrespective of their age (data not shown). This suggests that adult MSC display some, but not all senescence hallmarks.

### Adult MSC have a decreased capacity to maintain primitive CD34^+^CD38^−^ subpopulations and HSPC quiescence

**Figure 5:**
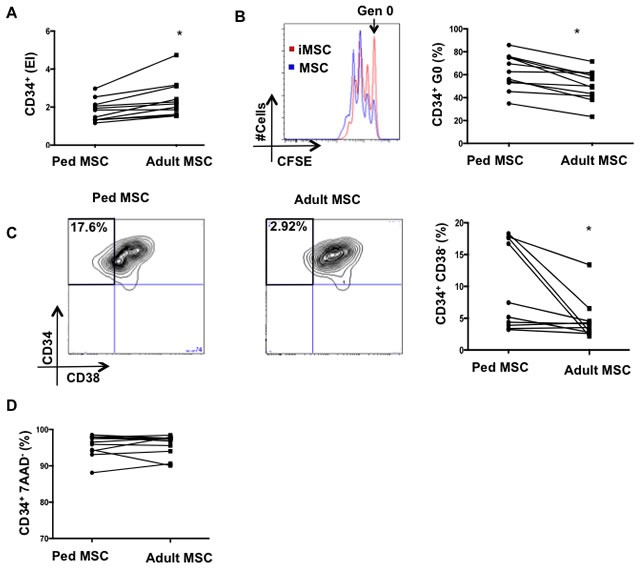
Loss of HSPC quiescence and primitive subpopulations in adult MSC-HSPC co-cultures **A.** CD34^+^ expansion, **B.** percentage of CD34^+^ in generation 0,**C.** frequency of CD34^+^/C38^−^ subpopulations, and **D.** viability were evaluated in HSC-MSC co-cultures (1:1 ratio) at day 4. MSC used were isolated from pediatric (< 16 years) or adult (> 40 years) donors. Mean of 10 independent experiments are reported where (*) represents *p* ≤ 0.05.

We next determined the impact of the chronological aging-induced senescent-like phenotype of MSC on their capacity to support HSPC. We co-cultured CD34^+^ cells from a single donor with MSC from adult or pediatric donors (Figure 5). We found that adult MSC induced higher overall CD34^+^ expansion compared to pediatric MSC co-cultures (expansion index 2.49±0.960 vs. 1.83±0.57; *p* = 0.04) (Figure 5A). The frequency of CD34^+^ cells in generation 0 was decreased in the adult compared to pediatric MSC co-cultures (49.57±13.89% *vs*. 61.20% ±15.46; *p* = 0.04) (Figure 5B). Furthermore, adult MSC have a reduced capacity to maintain CD34^+^CD38^−^ subpopulations compared to pediatric MSC (4.6±3.3 vs. 9.7± 6.8; *p* = 0.02) (Figure 5C). No differences were observed in CD34^+^ cell viability between adult and pediatric MSC cultures (*p* > 0.5) (Figure 5D).

Taken together, these results reveal important functional differences between adult and pediatric MSC. In particular, consistent with their senescent-like characteristics, adult MSC display a decreased capacity to maintain CD34^+^ quiescence and CD34^+^CD38^−^ subpopulations compared to pediatric MSC.

### IL-6 reduces HSPC quiescence in adult MSC co-cultures

**Figure 6:**
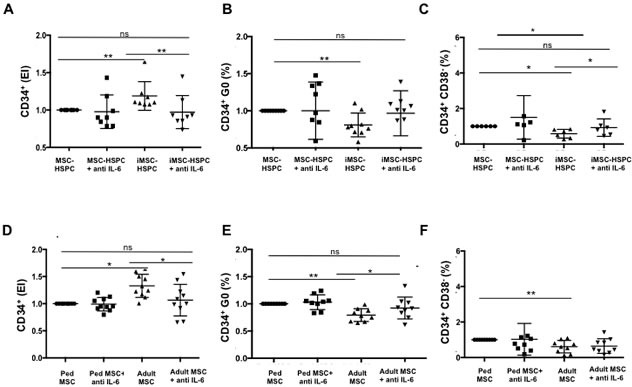
Increased production of IL-6 by senescent and aged MSC impairs HSPC quiescence **A.** CD34^+^ proliferation, **B.** percentage of CD34^+^ in generation 0, and **C.** frequency of CD34^+^CD38^−^ subpopulations were determined after 4 days in co-cultures with irradiated (5 Gy) and non-irradiated MSC in the presence or absence of an IL-6 neutralizing antibody (20ng/ml). Similar experiments were performed comparing the effect of IL-6 neutralization in adult and pediatric MSC-HSPC co-cultures **D.**-**F.** Results were normalized to 0 Gy control in **A.** -**C.** and Ped MSC condition in **D.**-**F.**. Mean value ± SD of 10 independent experiments are reported where (*) indicates *p* ≤ 0.05; (**) indicates *p* ≤ 0.01; (ns) indicates *p* > 0.05.

Given the elevated levels of secreted IL-6 by both irradiated and adult MSC and the relevance of IL-6 as a hematopoietin [21], we hypothesized that IL-6 played a role in promoting CD34^+^ expansion. To test this, we treated with an IL-6 neutralizing antibody iMSC and control co-cultures (Figure 6A-6C), and adult and pediatric MSC co-cultures (Figure 6D-6F).

The addition of anti-IL-6 to senescent MSC-HSPC co-cultures reduced the CD34^+^ expansion from 1.2±0.19 to 0.97±0.22 (*p* = 0.004 Figure 6A, values normalized to those of HSPC in non-irradiated co-cultures). IL-6 neutralization also increased the percentage of CD34^+^ cells in generation 0 (Figure 6B) and increased the frequency of CD34^+^CD38^−^ cells to levels that were similar to control conditions (Figure 6C). The addition of anti-IL6 to cultures however did not impact CD34^+^ viability (data not shown).

Similarly, blocking IL-6 in adult MSC co-cultures caused a significant decrease in CD34^+^ expansion (2.49±0.96 *vs* 1.93±0.67) (Figure 6D) and restored the proportion of CD34^+^ cells in generation 0 (49.57±13.89 *vs* 58.56±15.50) (Figure 6E). Those levels were not significantly different to those of pediatric co-cultures (expansion index: 1.88 ±0.58, percentage in generation 0: 61.20±15.46). The addition of IL-6 antibody to pediatric co-cultures however had no significant effect on CD34^+^ proliferation or quiescence. Blocking IL-6 tend to increase the percentage of CD34^+^CD38^−^ subpopulations in adult co-cultures and tend to decrease that subpopulation in pediatric co-cultures, albeit differences were not significant (*p* = 0.25) (Figure 6F). The addition of anti-IL-6 to adult and pediatric co-cultures had no significant effect on CD34^+^ viability.

These results suggest that increased IL-6 production by damaged or old MSC in co-cultures disrupts CD34 quiescence. The capacity to maintain CD34 quiescence, but not CD34^+^CD38^−^ populations, in adult co-cultures can be restored to pediatric levels by antagonizing IL-6.

## DISCUSSION

Hematopoietic stem cell function declines with age [22]. Given that signals from the bone marrow niche are key for normal hematopoiesis it has been proposed that age associated changes in the bone marrow niche contribute to HSPC aging [23]. Although there is an age-related decline in MSC immunopotency [24, 25]; it is unknown to what extent those age-related MSC chronological changes impact the microenvironment and HSPC function. This study provides direct evidence that adult MSC show hallmarks of senescence, in particular increased secretion of SASP factors, which alter HSPC homeostasis *in vitro*.

Our results indicate that much like other cell types, irradiation induces typical senescence hallmarks in MSC including growth arrest, SABG expression, and increase in cell size. These findings are consistent with previous studies looking at similar readouts in BM-MSC in response to irradiation [26, 27]. We further characterized MSC senescence by evaluating the presence of persistent DDR signaling and two conserved pro-inflammatory cytokines of the SASP. Consistent with a recent study that analyzed BM-MSC, we found that IL-6 and IL-8 secretion is increased in MSC in response to DNA damage (2-3 fold higher in iMSC compared to non-irradiated) [27]. The SASP influences cell migration, adhesion, and specifically in BM-MSC the upregulation of IL-6 in response to replicative senescence has been linked to a loss of stemness [28].

We next assessed the effects of MSC senescence on the MSC-HSPC crosstalk. Stem cell quiescence, protects HSC from accumulation of molecular damage, including the induction of replication associated DNA damage, oxidative-stress-induced damage, and telomere shortening [29]. MSC from various sources maintain CD34^+^ in an undifferentiated state *ex vivo* and this ability seems to be affected by MSC aging, as suggested by the fact that Terc^−/−^ MSC cells show decreased ability to maintain HSC [30]. We found that MSC support viable CD34^+^ cells preserving the CD34^+^CD38^−^ subset which has the highest reconstitution capacity [31]. Senescence decreased the MSC capacity to promote HSPC quiescence reducing the frequency of CD34^+^CD38^−^. This may reflect a shift from HSPC self-renewal towards HSPC proliferation and differentiation which ultimately could lead to stem cell pool replicative senescence and increase in genome aberrations [32].

To evaluate whether chronological aging recapitulates hallmarks of DNA damage induced MSC senescence, we compared the phenotype of adult and pediatric MSC and tested their HSPC supportive ability. Adult MSC have a “senescence-like” phenotype displaying only certain classical markers of cellular senescence. Compared to pediatric MSC, the adult MSC size is larger [33] and they secrete 4-times higher levels of IL-6 and IL-8. Furthermore, consistent with previous reports, other markers of senescence, specifically SABG activity was absent in early passage adult and pediatric MSC [19, 20].

While adult MSC did not display all of the classical features of cellular senescence, we demonstrate for the first time that adult MSC functionally behaved similarly to senescent MSC. Like irradiated MSC co-cultures, adult MSC promoted CD34^+^ expansion while retaining fewer CD34^+^ in generation 0 and decreased the frequency of primitive CD34^+^CD38^−^ cells. These results would favor the use of pediatric MSC over adult MSC for the *ex vivo* expansion of CD34^+^ cells. Alternatively, our results also raise the possibility that the manipulation of senescent MSC *in vivo via* pharmaceutical inhibition of SASP factors like IL-6 or through targeted elimination of these cells, as is now performed in mouse models, could be used in the context of natural aging or of premature aging syndromes [6, 8, 34, 35, 36, 37].

In the context of aging, we hypothesize that an altered HSPC-MSC cross-talk can promote HSPC proliferation resulting in a functional exhaustion of the hematopoietic system. This negative impact of microenvironment senescence or aging on the regulation of HSPC quiescence was previously suggested following an *in vitro* evaluation of MSC replicative senescence demonstrating that early passage MSC favor HSPC self-renewal whereas culture-aged late passage MSC favor differentiation and expansion of HSPC [38]. *In vivo*, the aging of the stem cell niche in a different context has also been shown to disrupt satellite muscle cells quiescence leading to a decline in regenerative capacity [39]. Furthermore, multiple myeloma -MSC derived from deceased patients displayed features of cellular senescence such as SABG activity and increased their hematopoietic support capacity, suggesting that besides their role in bone marrow aging, senescent and dysfunctional MSC can also influence cancerous tissue microenvironments [40].

Overall, our results suggest that the decrease in HSPC quiescence and CD34^+^CD38^−^ subpopulations is linked to the increased in senescence-associated IL-6 production by senescent/adult MSC. A neutralizing antibody against IL-6 rescues HSPC quiescence and restores the frequency of the CD34^+^CD38^−^ subpopulation to levels similar to those in control/pediatric co-cultures. Thus, IL-6, a potent hematopoietin that promotes myeloid differentiation and suppresses lymphoid differentiation, could induce HSPC differentiation at the expense of self-renewal and quiescence. This could contribute to the defective maintenance of primitive stem cells in the context of age-associated pathologies and the increased proliferation of cancer cells potentially using this defective microenvironment as a source of growth signals.

Overall the findings from this study demonstrate that age related changes in MSC directly affect critical properties of HSPC. Both senescent MSC and adult MSC produce elevated levels of IL-6 which decreases HSPC quiescence and reduce CD34^+^CD38^−^ subpopulations. Antagonization of IL-6 ‘rejuvenates’ adult and senescent MSC, making them functionally similar to pediatric MSC.

## MATERIALS AND METHODS

### Patients

This study was approved by the McGill University-Ethics Board. Written consent was obtained from all participants. Adipose tissue samples were obtained from 19 adult donors undergoing cardiovascular surgery and 17 pediatric donors undergoing orthopedic surgery. Table 1 summarizes the demographic information of all study participants.

**Table 1:** Demographics and clinical features of the adult and pediatric MSC donors

	Adult donors (*n* = 19)	Pediatric Donors (*n* = 17)
*Demographics*		
Sex (% female/male)	36/64	53/47
Age (mean ± SD years)	59.4±12.9	13.9±2.8
*Surgical Procedure (%)*		
Cardiovascular	100	-
Orthopaedic	-	100
*Clinical Characteristics (%)*		
Tobacco Use	40	-
Diabetes	42	-
*Medications (%)*		
Statins	64	-
Aspirin	72	-
Metformin	36	-

### Isolation and characterization of MSC

MSC were isolated from adipose tissue as previously described [41] with minor modifications. Briefly following enzymatic digestion, cells were plated in 75cm^2^ flasks (1g tissue/75cm^2^ flask) and cultured at 37°C in a 5% CO_2_ incubator. Adherent cells were maintained in low glucose DMEM (Wisent Inc, cat 319-010-CL) supplemented with 10% MSC Qualified FBS (Gibco, ref 12662-029) and 1% Pen Strep (Gibco, ref 15140-122) until they reached 80% confluence, at which point they were trypsinized (Corning Cellgro, ref 25-053-CI) and reseeded at a density of 5000 cells/cm^2^. Isolated MSC were characterized fulfilling the minimal criteria proposed by the International Society for Cellular Therapy (ISCT) [12]. Cell surface markers were evaluated by FACS at the end of passage 2 with fluorescence conjugated anti-human antibodies all from BD biosciences: CD73-PE, APC-CD34, FITC-CD90, CD105-PECYP CY5.5, CD19-APC, HLADR-APC, CD14-PERP CY5,5, and CD45-FITC. At passage 3, MSC tri-lineage differentiation was tested using the adipogenesis/chondrogenesis differentiation kit (StemPro Cat # A1007101), and osteogenesis differentiation kit (StemPro Cat # A1007201) according to the manufacturers instructions. After 3 weeks, cells were fixed with 3.7% paraformaldehyde and osteoblasts were stained with Alizarin Red S (Alizarin Red S, Certified, Electron Microscopy Sciences, Product #10360(EM)), chondrocytes with Safranin-O, (Safranin O, Certified, Electron Microscopy Sciences, Product # 20800 (EM)) and adipocytes with Oil Red (Oil Red O, Electron Microscopy Sciences, Product # 19056 (EM)).

### CD34-based HSPC isolation

HSPC were isolated from non-mobilized peripheral blood by magnetic cell sorting (auto-MACs) using the CD34 Microbead Kit (Miltenyi Biotec # 130-046-702) as previously described [42, 43].

### MSC-HSPC co-cultures

At passage 3 MSC were trypsinized and seeded in a 96 well plate (9000 cells/cm^2^). Twenty-four hours later the medium was changed to a serum free medium (Stem Span H3000, Stem Cell Technologies, Vancouver, BC). After 2 days of culture in serum free medium, freshly isolated CD34 cells were stained with carboxyfluorescein succimmidyl ester (CFSE) as previously described [42, 43], suspended in Stem Span H3000 (Stem Cell Technologies, Vancouver, BC), added to the allogeneic MSC cultures in a 1:1 ratio and co-cultured for 4 days.

### IL-6 neutralization

A neutralizing antibody against IL-6 (RD Systems cat # MAB206) or IgG isotype (control) was added to co-cultures at day 0 and day 2 at a concentration of 20ng/ml.

### Flow cytometry analysis

At day 4 of co-culture, HSPC and MSC were trypsinized and stained with CD34-APC, CD38-PE, and 7AAD. Flow cytometry (BD LSR Fortessa) data was analyzed with the FlowJo analysis software (Tree Star, USA). The fold-expansion of the overall HSPC culture (expansion index) was calculated with FlowJo. MSC size was expressed as the median signal intensity of FSC-A readings.

### MSC irradiation

MSC were treated with 5 Gray (Gy) or otherwise indicated doses of gamma irradiation using GammaCell 22 and placed in a 6 well plate (5000cells/cm^2^) in complete DMEM. Four days post irradiation the cells were co-cultured with HSPC (see above).

### Immunofluorescence

Cells were seeded on glass slides (0.70 cm^2^ or 1.70cm^2^) and fixed at indicated times in 10% fixation last 10 minutes followed by permeabilization last 30 minutes in 0.5% Triton X-100 (Sigma cat#93443) in phosphate-buffered saline (PBS). Subsequently, cells were incubated in blocking solution (1% BSA, IgG free, protease free, 4% Normal Donkey Serum (Jackson ImmunoReaseach cat#001-000-162, Sigma cat#D966) for 60 min prior to incubation with primary antibodies over night at 4°C (53BP1: Novus biologicals cat#NB 100-304, PML: Santa Cruz cat#sc-9862, γH2AX: Upstate cat#05636). Cells were then washed with PBS and incubated with indicated secondary antibodies for 60 minutes (Life technologies Alexa Fluor anti-goat/mouse/rabbit 488nm/594nm). The slides were finally washed, counterstained with Hoechst and mounted with Vectashield (Vector Laboratories cat#H-1000). Images were acquired using a Axioobserver Zi Zeiss Scanning Microscope and processed/analyzed using Photoshop CS5 and the Axiovision software (Assay builder).

### Senescence-associated beta-galactosidase (SABG) staining

Non-irradiated (control) and 5Gy irradiated MSC were counted and plated 24h post irradiation. SABG activity was assessed 8 days post-irradiation using previously described protocols [19].

### ELISA

MSC were plated in 96 well plates (2500 cells/well) and supernatants were collected at day 2-4 post seeding in serum free medium (Stem Span H3000, Stem Cell Technologies, Vancouver, BC) and stored at −80C. Quantification of IL-6 and IL-8 was done with ELISA (Human IL-6 kit (BD OptEIA cat#555220) and Human IL-8 kit (BD OptEIA cat#555244) according to the manufacturers protocol. ELISAs were performed in triplicates and results were normalized to cell number (DRAQ5 intensity).

### DNA staining (LICOR)

Cells were seeded in 96-well plates (Clear black, Greiner Bio one 655090) at 1000 cells per well and cultured/treated as indicated. At selected experimental points, plates/cells were washed, fixed, and incubated with a DRAQ5 solution (5mM Cell Signaling #4084, diluted 1:10000). Following the final wash the plates were scanned using a LI-COR Odyssey system. The data from digitized signal intensity was processed using the Trim Mean option in the Image Studio 3 software.

### Statistical analyses

Summary data is presented as mean ± standard deviation. Wilcoxon matched-pairs signed rank test was used to assess differences in the MSC irradiation experiments, whereas Mann-Whitney test was used for the comparisons between the adult and pediatric MSC. All analyses were performed using the GraphPad Prism software (Graph-Pad, San Diego, USA). All hypotheses tests were 2-sided and significance was set at the 0.05 level.

## SUPPLEMENTARY MATERIAL FIGURE


