# Supplementary material for: Increased IL-6 secretion by aged human mesenchymal stromal cells disrupts hematopoietic stem and progenitor cells' homeostasis
Source: Oncotarget. 2016 Feb 24;7(12):13285–96. doi: 10.18632/oncotarget.7690 (PMC4924641; doi:10.18632/oncotarget.7690)
Supplement: Supplementary file 1 [file oncotarget-07-13285-s001.pdf]

Increased IL-6 secretion by aged human mesenchymal stromal cells disrupts hematopoietic stem and progenitor cells' homeostasis

Supplementary Material

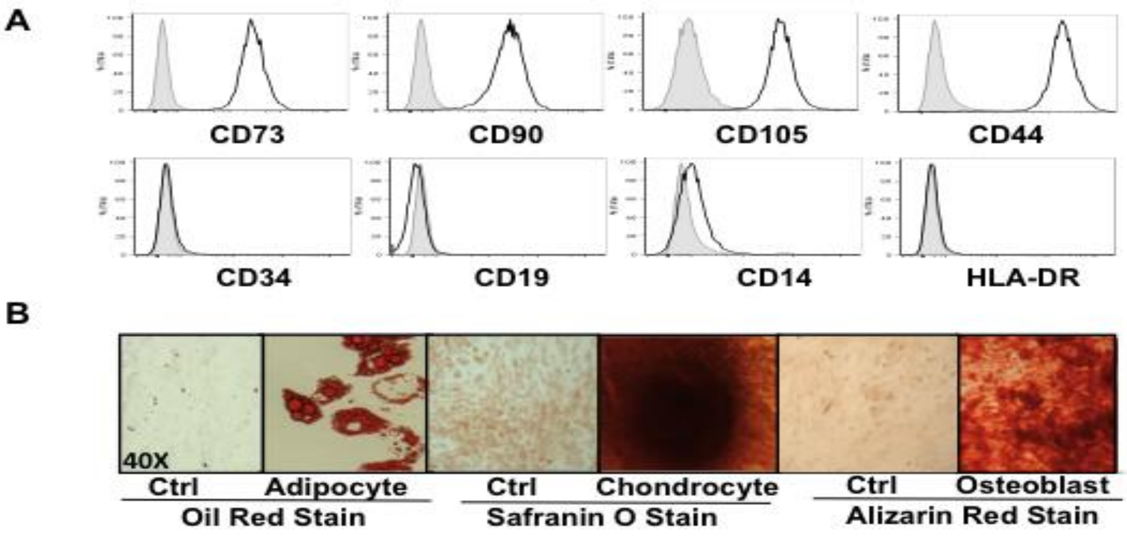

Supplementary Figure 1 Characterization and validation of adipose derived human MSC.

(A) The expression of CD73, CD90, and CD105 (positive biomarkers), and lack of expression of CD34, CD19, CD14, HLA-DR (negative biomarkers) was assessed by flow cytometry in every sample at passage 2. CD44, a marker for pluripotency highly conserved across species, was included in the evaluation. In all the samples tested and used for this study, >95% of the cells expressed the positive and <5% expressed the negative biomarkers. Representative example (black histograms) with negative controls (unstained samples – grey histograms).

(B) All MSC samples were functionally validated for their adherence to plastic and their capacity to differentiate into osteoblasts, chondrocytes and adipocytes under appropriate conditions. Differentiation analysis of a representative MSC sample is displayed.
